# Editorial: A new frontier for traditional medicine research—Multi-omics approaches

**DOI:** 10.3389/fphar.2023.1203097

**Published:** 2023-05-09

**Authors:** Xianjun Fu, Kah Keng Wong, Yiider Tseng

**Affiliations:** ^1^ Shandong University of Traditional Chinese Medicine, Jinan, China; ^2^ Qingdao Key Laboratory of Research in Marine Traditional Chinese Medicine, Shandong University of Traditional Chinese Medicine Qingdao Academy of Traditional Chinese Medicine, Qingdao, China; ^3^ Universiti Sains Malaysia, School of Medical Sciences, Kota Bharu, Malaysia

**Keywords:** traditional medicine, multi-omics approaches, TCM syndrome, therapeutic mechanism of TCM, biological processes

Traditional medicine accumulates cultural theories, beliefs, and experiences that are unique to different populations, whether scientifically explicable or not. It is used in the maintenance of health as well as in the prevention, diagnosis, improvement, or treatment of physical and mental illness (Che, et al., 2017). However, the lack of scientific exploration, empirical diagnosis, and rigorous therapeutic strategy hinders greater access to better understanding these healthcare services (Patwardhan and Patwardhan, 2005). To address these issues, multi-omics experimental designs analyze pharmacological action and biological mechanisms at the molecular level by integrating multiple omics such as the genome, transcriptome, proteome, metabolome, epigenome, and microbiome, thus shifting the paradigm of “single omic” research (Menyhárt and Gyorffy, 2021). Multi-omics provides a powerful approach to gathering further hereditary information of the organism and illustrating a wide range of biological phenomenon, such as identifying the bioactive components, action targets and biological pathways of various traditional Chinese medicine. This integration of modern scientific insights with the role of traditional Chinese medicine in a variety of diseases, including cardiovascular diseases, infectious diseases, metabolic diseases, and neurodegenerative diseases, (Zhu et al.).

In this Research Topic, we have collected twelve research papers and review articles focusing on multi-omics research related traditional Chinese medicine to biological mechanisms, and the complex biological processes in traditional therapies.

This Research Topic comprises a variety of article types, including animal experiments (6 papers), clinical research (2 papers), reviews (2 papers), meta-analysis (1 paper), and comparative metabolic profiling and its substituents in traditional Chinese medicine (1 paper). The related Multi-Omics Approaches range from proteomics to metabolomics, intestinal microbiomics, and lipomics. These articles cover diseases such as COVID-19, cardiomyopathy, triple-negative breast cancer, depression, fatty liver related to metabolic dysfunction, coronary heart disease, chronic liver disease, and stroke. Traditional Chinese Medicine (TCM) syndromes, such as phlegm stasis and damp heat, as well as research on toxicity and metabolic substances of TCM, are also included. The 108 authors who contributed to these papers are from 43 institutes located in China.

Six contributions in this Research Topic focus on the effect of traditional Chinese medicine on different diseases using a multi-omics research approach. The first two contributions address depression. Qiao et al. evaluated the effects of Tibetan medicine, metacinnabar (β-HgS) combined with imipramine or sertraline (SER) on depression-like symptoms in mice. They revealed that β-HgS promotes the antidepressant effect of SER on depression-like behavior in mice by promoting glucocorticoid receptor (GR) expression and neuronal proliferation in key hippocampal subregions. Liang et al. evaluated the antidepressant efficacy of Yang-Xin-Jie-Yu Decoction (YXJYD) in a chronic unpredictable mild stress (CUMS)-induced depression rat model and investigated the underlying mechanisms by using metabolomics and intestinal microbiomics methods. They identified the pathway of the tricarboxylic acid cycle (TCA cycle) and propanoate metabolism as the regulated target of YXJYD on host-microbiome interaction. He et al. found that *Poria cocos* extract can affect metabolic dysfunction-associated fatty liver disease via the FXR/PPARα-SREBPs pathway. Wang et al. presented a study revealing that Dandelion extract inhibits triple-negative breast cancer cell proliferation by interfering with glycerophospholipids and unsaturated fatty acids metabolism. Liu et al. revealed that astragaloside IV significantly promotes pharmacological effect of *Descurainia sophia* seeds on isoproterenol-induced cardiomyopathy in rats by complementarily reversing myosin motor MYH6/7, and further downregulating NPPA and MYL4. Chien et al. presented a systematic review and meta-analysis of randomized controlled trials about the therapeutic effects of herbal-medicine combined therapy for COVID-19.

Three contributions focus on using the multi-omics research approach to explore the biological basis of TCM syndrome. Yang et al.revealed that coronary heart disease with phlegm and blood stasis syndrome is characterized by low levels of FOS, AP-1, CCL2, CXCL8, and JNK1, and elevated levels of PTGS2 and CSF1 by using a strategy that integrated RNA-seq, DIA-based proteomics, and untargeted metabolomics on 90 clinical samples. Pan et al.found distinct common signatures of gut microbiota associated with damp-heat syndrome, a status of disharmony that often occurs when dampness binds with heat evil, in patients with different chronic liver diseases. Liu et al. summarized and highlighted the latest significant progress in the crucial value of applying multi-omics approaches to reveal TCM syndromes of stroke in a new horizon.

Two contributions focus on the toxicity and metabolic substances of TCM. Miao et al. revealed novel insights into the mechanism of hepatotoxicity induced by *Tripterygium wilfordii* multiglycoside in mice via proteomics analysis and demonstrate that the gut-liver axis may play a vital role in the progression of *Tripterygium wilfordii* multiglycoside-induced hepatotoxicity. Guo et al. comprehensively profiled the metabolites in wild Chinese Cordyceps species from Naqu (NCs) and Yushu (YCs) and their substituents including artificially cultivated Cordyceps species (CCs) and mycelia, by using liquid chromatography-tandem mass spectrometry (LC-MS/MS)-based metabolomics analysis. They analyzed quantitatively seventy amino acid-relevant metabolites in four samples for the first time.

The remaining contribution, Zhu et al. presented a review of multi-omics approaches for an in-depth understanding of the therapeutic mechanism of TCM. They evaluated and compared several TCM databases for storing multi-omics data in terms of completeness and reliability.

As a summary, we believe that the papers we have collected contain a wide range of multiple-omics methods which can help us systematically understand the biological basis of TCM syndromes, the mechanisms by which TCM treats diseases, as well as the spectrum-effect relationship of TCM components.
